# Assessing the predictors for training in management amongst hospital managers and chief executive officers: a cross-sectional study of hospitals in Abuja, Nigeria

**DOI:** 10.1186/s12909-018-1230-2

**Published:** 2018-06-14

**Authors:** Ogbonnia Godfrey Ochonma, Stephen Ikechukwu Nwatu

**Affiliations:** 1 Department of Health Administration and Management, Faculty of Health Sciences and Technology, College of Medicine, University of Nigeria, Enugu Campus, Enugu State, Nigeria; 2 Administration Unit, M&M Hospital, the fertility and IVF Center, FCT, Abuja, Nigeria

**Keywords:** Hospitals, Managers, Training, Informal, Formal, Need, Abuja, Nigeria

## Abstract

**Background:**

There is a compelling need for management training amongst hospital managers in Nigeria mostly because management was never a part of the curricula in medical schools and this has resulted in their deficiencies in effective policymaking, planning and bottom line management. There has been no study to the best of our knowledge on the need and likely factors that may influence the acquisition of such training by hospital managers and this in effect was the reason for this study.

**Methods:**

Data for this study came from a cross-sectional survey distributed amongst management staff in twenty five (25) hospitals that were purposively selected. One hundred and twenty five (125) questionnaires were distributed, out of which one hundred and four (104) were answered and returned giving a response rate of 83.2%. Descriptive and Inferential statistics were used to summarize the results. Decisions were made at 5% level of significance. A binary logistic regression was performed on the data to predict the logit of being formally and informally trained in health management. These statistical techniques were done using the IBM SPSS version 20.

**Results:**

The result revealed a high level of formal and informal trainings amongst the respondent managers. In formal management training, only few had no training (27.9%) while in informal management training, all had obtained a form of training of which in-service training predominates (84.6%). Most of the administrators/managers also had the intention of attending healthcare management programme within the next five years (62.5%). Socio-demographically, age (*p* = .032) and academic qualification (*p* < .001) had significant influence on training. Number of hospital beds (*p* < .001) and number of staff (*p* < .001) including managers’ current designation (*p* < .001) also had significant influence on training.

**Conclusion:**

Our work did establish the critical need for both formal and informal trainings in health management for health care managers. Emphasis on training should be directed at younger managers who are the least likely to acquire such trainings, the smaller and private hospitals who are less likely to encourage such trainings amongst their staff and the least educated amongst health managers.

**Electronic supplementary material:**

The online version of this article (10.1186/s12909-018-1230-2) contains supplementary material, which is available to authorized users.

## Background

A hospital chief executive and manager designated as chief medical director (CMD) in Nigeria is most of the time a physician and shoulders the responsibility of managing his/her institution in a way that assures not just good clinical practice, but excellence in strategy, financial prudence and good human resource management as well. The trend in Nigeria according to Adindu in management training and health managers [1] shows that many health managers lack adequate training in providing strategies that enables prudent and effective management of all the available resources and amenities for the proper functioning of the hospitals. This according to the author [1] is because healthcare management was never a part of the curricula in medical schools. Clinicians in Nigeria as health managers in view of the numerous challenges of the health sector, and poor health situations require training that prepares them for effective policymaking, planning, decision-making, organizing, coordinating and effectively utilising resources for service delivery, but that has not been the case in Nigeria concludes the author [1]. Health sectors around the world are typically labour intensive depending on diverse workers to provide services and to support system operation as observed by Adindu et; al in training human resources for twenty-first Century Nigeria Health Sector [2]. The quality of training affects performance of health workers, health of people, and health of the nation [2]. Human resource management is the integrated use of systems, policies, and practices that provide a range of functions needed to plan, produce, deploy, manage, train, support and sustain the health workforce according to Adindu as observed by Marsden et; al [2, 3]. Health training institutions prepare health workers to perform these functions as indicated by Adindu and WHO [2, 4]. However, countries with critical shortages and imbalance of health workers often lack the technical capacity to identify and assess crucial policy issues of the health workforce [2, 4]. Health professionals function both as clinicians and managers in Nigeria, yet training in health services management missing in the curricula in medical schools is critical to bridging the knowledge gap for health professionals to perform management functions effectively [2, 4].

Health management involves technical and social processes that managers carry out to achieve organisational objectives through effective and efficient use of health resources, within social, cultural, political, and economic realities of the context [2]. However, regardless of context, application of effective management principles and leadership are necessary, which depend on skills and competencies, orientation and attitude of health professionals who become managers at different levels. Every health care professional requires skills to develop individual action plans, participate in operational, departmental, and strategic planning [2].

The country has huge stock of health care professionals, with 58,325 medical doctors, 14,353 pharmacists, 137,198 nurses, 93,743 midwives, 2426 dentists, 14,887 medical laboratory scientists, 924 radiographers, 2131 optometrists, and about 40,491 community health workers [1, 5]. Yet, quality of service is generally poor, health indices among the poorest in Africa; with under five mortality of 186 per 1000 live births, and maternal mortality ratio of 1100 per 100,000 live births as Adindu observed of the WHO recommendations [1, 6].

It is our take that health managers should apply excellence in management practices in not just clinical practice but also in tactical management strategies in human resource and financial management to achieve good results given the poor indices of our health system in general and hospitals in particular. Often health managers receive patchy training in management; a few continually attend training for benefits derived from being away from office rather than knowledge; and those with substantial knowledge and skills are unable to apply such within unstructured and uncoordinated management system [1].

Continuing education and training have become part of the ongoing processes of organisational learning and permanent change, employee evaluation, and career development as observed by Zorica et; al in training hospital managers for strategic planning and management [7, 8]. In addition, they are essential tools for managers to improve their management skills and to learn new skills [7–11]. Training can be defined as the systematic acquisition of skills, rules, concepts, or attitudes, which result in improved performance [7, 12]. There is an increasing demand for formal and informal training programs in health organisations, especially for physicians in leadership positions who need to acquire managerial and leadership skills. In some hospitals, significant resources are devoted to educate the institution’s managers [7, 13]. Training is one of the most important components in any organisation’s strategy, and evaluation is an essential part of the training system [7, 14].

Many corporate business owners and managers give little thought to staff education. They do not think it is part of their role, or if they do, they never have time to get around to it. For physicians in practice, another factor may contribute to a lack of involvement in staff development as advanced by Dean et; al in essentials of staff development [15]. Physicians may reason that their energy, not to mention their years of education and training, should be focused on patient care, research, and staying abreast of clinical advancements, whereas staff education should fall under the category of personnel and be the responsibility of practice managers [15]. Yet, staff education is a role they must oversee especially as it pertains to management of resources and formulation of strategies that enables overall functioning of all facets of the hospital [15].

Many managers have never had any formal management training [15]. They became managers because they excelled in their work, but the skills that made them the best nurses, the most efficient coders, good physicians or top-notch office organizers do not necessarily make them first-rate managers [15]. But good management skills are critical, because employees often decide to stay in jobs or leave them depending on how their bosses treat them, not because of the organisation for which they work [15] .Recommends setting an expectation that all managers in hospital practice receive management training.

Research on district hospital management in Kenya suggests that senior managers are often not well prepared for management role although there are current efforts to build skills in these areas through “management training” as indicated by Nzinga et; al in service delivery in Kenyan district hospitals [16–18]. Previous work in Kenyan public hospitals has revealed leadership gaps and poor communication between senior administration and lower cadres as an impediment to achieving better practice [15, 19, 20]. Management training for senior health professionals has been recognized as a priority and is now being provided as suggested by *Report on Management and Leadership Development Gaps for Kenya Health Managers* [21].

In Portugal, the shortage of skills remains a key obstacle for innovation, particularly in hospitals pursuing radical and disruptive changes as indicated by Dias et; al in narrowing the skill Gap for innovation [22, 23]. Therefore, it has become important to ensure that a skilled workforce is present for delivering high quality health services to strengthen the health system in Portugal, as well as across Europe. Such emphasis on skills and innovation is reflected by several efforts to bridge and narrow the current skills and productivity gap. Hospitals have long focused on the development of skilled health professionals through continuous efforts in training and education [22, 23].

The chief medical directors (CMDs) reports a news paper article in Nigeria must be trained in the business of medical administration [24]. The whole problem of the poor performance of CMDs in many public institutions stems from their lack of preparedness for the position. Many are entrusted with powers beyond their mental capabilities simply because of ethnicity, tribal, religious or political affiliations. Future CMDs must be trained in business administration to a high level of competence if we are to make serious progress in building hospitals of the future as indicated by the paper [24].

Nothing in a doctor’s training prepares him for financial management. ‘We are simply not trained to look after money like lawyers, for example. So, stop trusting doctors with the finances of the hospital’ replies a doctor [24]. However, there is some emerging evidence from the United States that hospitals run by doctors with hospital administration qualifications appear to do better [24]. The job description of the chief medical director (CMD) should emphasize medical management of the staff and medical service delivery. This should also include provision of services and hospital development in line with local needs concludes the paper [24].

We have explored to this time, the need for training of health managers in management strategies to enable them shoulder the enormous responsibilities of the hospital sector in Nigeria. We have also explored internationally, the recognized need to improve on hospital performance through the acquisition of management skills by the administrative and clinical staff officers.

There is no gain in saying that hospital chief executive officers and line managers when equipped with the right blend of management trainings are likely to be successful management strategists both in human resource management and financial prudence of their facilities. There has also been established the need for management trainings for hospital managers to excel in their duties. We have also observed through the review of the literature that there has been no study to the best of our knowledge on the likely factors that may influence the acquisition of managerial competence and trainings by hospital managers and chief executive officers.

This study in effect, was therefore instituted to explore the likely factors that may influence hospital managers and chief executive officers to acquiring trainings and competencies in management strategies that will help improve their performance in hospital management. The result of this work, it is hoped will enable the structuring of strategies to enable the acquisition of managerial trainings to help better the performance of hospital managers in Nigeria.

## Methods and subjects

Data for this study came from a cross-sectional survey using self-administered questionnaire distributed among management staff in twenty five (25) hospitals that were purposively selected. The criteria for selection were that each of the hospitals must be at least twenty (20) bedded and employs at least twenty five (25) persons. A pre-tested structured self-administered questionnaire was used during the period March to April 2015 to collect the preliminary data from each respective respondent. The pre-testing of the instrument was done sixteen weeks earlier before the commencement of this study to strengthen the validity and reliability of the questionnaire. The responses from the pre-testing contributed to the restructuring of the questions to measure exactly what was intended. More so, the questions were first translated into the local language (*Igbo*) and back to English language to strengthen their reliability. The questionnaire which was developed by the authors after careful observation of previous works [1, 2] on the subject was used by the managers in self-assessing their training needs. Through the pre-testing, we demonstrated both construct and criterion validities in that the instrument was actually improved to measure what was intended for it to measure (construct validity) and we also made sure our measurement was related to our outcome (criterion validity). Before this study was undertaken, most hospital managers in Nigeria were hired into their present positions as managers without the relevant hospital management certification and that affected their performance. But recently, certifications and relevant degrees are being offered by Nigerian universities to enhance the performance of hospital managers. Our study in essence was theoretically designed to measure the degree to which our study population (hospital managers) will undertake programmes in health management (also referred to here as the construct related validity) to enhance their performance as hospital managers using age, gender, academic qualification, hospital type, hospital beds, number of hospital staff employed in the hospital, current designation, experience in hospital management and declared intention to attend a programme in the future as the criteria and predictive values (also referred to here as the criterion related validity). In a well functioning institution, the results of this analysis could be used as some of the criteria for hiring management staff in the hospital depending on the goal(s) of the institution. It is our take (theoretically) that a manager’s likelihood to undertake a health management academic programme(s) in the future to enhance his performance would be influenced by the criteria as mentioned above. In essence, our study examined how the criterion related validity (age, gender, academic qualification etc.;) could be used to predict the probability of our study population undertaking academic programmes in health management (construct validity) in the future to enhance their performance as hospital managers.

### Reliability results

The reliability result indicates an understanding of the contents of the questions between the managers and the researchers as the responses to both the Igbo and English versions of the questions were the same from different sets of respondents. The responses to the questions from smaller groups produced same results over time. Questions that were confusing and did not make any sense to the respondents were either amended or discarded. This indicates that the study could be replicated with similar population groups producing similar results. The time span between the two administrations of the instrument was 8 weeks apart.

The self assessed training needs by the managers contained in the questionnaire include:

(Please see Additional file 1 as uploaded).

Have you had any formal training in healthcare management?

- Certificate ()
- Diploma ()
- Degree ()
- None ()
- Others (please specify) () -------------

Have you had any informal training in healthcare management?

- In-service training (workshops, seminars etc) ()
- Mentoring ()
- Non certified courses ()
- Others (please specify) () -------------

Do you intend to attend any health care management programme within the next five years?

- Yes ()
- No ()

If yes please specify -----------------.

Hospitals in the federal capital territory (FCT) Abuja, Nigeria with a minimum of 20 beds and 25 staff as employees were used in the study with the surveyed staff being designated as Hospital Director, Hospital Manager, Hospital Administrator, Hospital Chief Executive Officer or Chief Medical Director. Those provided with the questionnaire were also heads of units responsible for the day to day administration and operation of hospital amenities with a minimum of diploma or bachelor’s degree (or equivalent) obtained in any academic discipline. Questionnaires were distributed directly to the respondents. One hundred and twenty (125) questionnaires were distributed, out of which one hundred and four (104) were answered and returned giving a response rate of 83.2%.

### Ethics approval and consent to participate

A local ethics committee (University of Nigeria ethical review committee) ruled that no formal ethics approval was required in this particular case and study. Permissions were eventually gotten from the various (25) hospitals’ Chief Medical Directors (CMDs) offices to conduct the research in each of the hospitals. Consent to participate in the study was verbal. We used this method of consent participation because it was convenient and immediate as opposed to written consent. The respondents were assured of their confidentiality and were provided with the choice of not partaking in the study if they so wished. The research was conducted according to Helsinki declaration and local legislations.

The following hospitals’ Chief Medical Directors’ (CMD’s) offices permitted that this work be conducted in each of the hospitals below.

1. ISA Premiere Hospital, Jabi, Abuja
2. Garki Hospital, Garki Abuja
3. M&M Hospital, Karshi, Abuja
4. Kelina Hospital, Gwarimpa, Abuja
5. Zanklin Medical Center, Abuja
6. Primus supper Specialty Hospital, Karu, Abuja
7. RUZ Medical and Diagnostic Center, Abuja
8. St Francois Medical Center Abuja
9. Asokoro General Hospital, Abuja
10. Alhassan Hospital, Abuja
11. Amana Medical Center, Abuja
12. First Hospital and Maternity, Abuja
13. Horizons Medical center, Abuja
14. Ideal Hospital, Abuja
15. Iduna Specialists Hospital, Abuja
16. Kings Care Hospitals, Abuja
17. University of Abuja teaching Hospital, Gwagwarada, Abuja
18. Federal Staff Clinic, Abuja
19. Wuse general Hospital, Abuja
20. National Hospital, Abuja
21. Cedar Crest Hospital, Abuja
22. Silver Fountain Hospital,Abuja
23. Abuja Unity Hospital and Maternity, Abuja
24. Bio Royal Hospital, Abuja
25. Corner Stone Specialists Hospital, Abuja

### Method of data analysis

The collected data was subjected to both descriptive and inferential statistics. Descriptive statistics- frequency, percentage, mean and standard deviation were used to summarize the items of the questionnaire. Inferential statistics- Chi-Square Test of Association and Fishers Exact Test were used to determine the influence of socio-demographic, hospital and health management characteristics on formal training in health management. However, Fishers Exact Test was specifically used at Chi-Square assumption violation. Decisions that were made on all inferential statistics were at 5% level of significance. A binary logistic regression was also performed on the data to predict the logit of being formally training in health management. The socio-demographic, hospital and health management characteristics served as the predictors while the formal training status (that is, whether formally trained or not) served the predicted variable. These statistical techniques were done using the IBM SPSS version 20. (Please see Additional file 2 as uploaded).

## Results

Table 1 displays the socio demographic and hospital characteristics of the managers. Majority of them were aged between 35 and 45 years (52.9%). There were more males (63.5%) than females (36.5%) amongst them. A little above thirty four (34.6%) percent had bachelor’s degree, 27.9% had post graduate degree while 33.7% had master’s degree. Most of them were either in the private hospital (42.3%) or government hospital (39.4%). In number of staff, those who had 50–100 staff (34.6%) were more followed by those who had 25–50 staff (33.7%) while in the number of beds, it was those who had 50–100 beds in their hospital (50.0%) were in majority.

**Table 1:** Managers’ socio demographic and hospital characteristics *n* = 104

	Frequency	Percent
^+^Age		
25–35 years *(< 45 years)*	14	13.5
35–45 years *(< 45 years)*	55	52.9
45–60 years *(> 45 years)*	35	33.7
Gender		
Male	66	63.5
Female	38	36.5
^a^Academic Qualification		
Bachelor’s degree *(First degree)*	36	34.6
Post graduate diploma *(Higher degree)*	29	27.9
Master’s degree & higher *(Higher degree)*	35	33.7
Others	4	3.8
^+^Hospital type		
Private *(Non-governmental)*	44	42.3
Government *(Governmental)*	41	39.4
Non-governmental *(Non-governmental)*	5	4.8
Faith based *(Non-governmental)*	14	13.5
^a^No. of staff in the hospital		
Below 25 *(< 25 staff)*	20	19.2
25–50 *(25–50 staff)*	35	33.7
50–100 *(> 50 staff)*	36	34.6
100 and above *(> 50 staff)*	13	12.5
No. of hospital beds		
25–50 beds	26	25.0
50–100 beds	52	50.0
> 100 beds	26	25.0

^a^implies variables in which some groups were merged to enhance further analysis

Table 2 displays the managers’ characteristics with regards to healthcare management. Twenty four (24.0%) percent of them were administrative officers, 18.3% were hospital administrators and as well CEO/hospital directors while 39.4% were medical directors. Majority of them had 3–10 years hospital management experience (47.1%). In formal healthcare management training, only few had no training (27.9%) while in informal healthcare management training, all had obtained a form of training of which in-service training predominates (84.6%). Most of the administrators/managers also had the intention of attending healthcare management programme within the next five years (62.5%).

**Table 2:** Managers’ characteristics in healthcare management n = 104

	Frequency	Percent
Current designation		
Administrative officer	25	24.0
Hospital administrator	19	18.3
CEO/Hospital director	19	18.3
Medical director	41	39.4
Experience in hospital management		
Less than 2 years	24	23.1
3–10 years	49	47.1
> 10 years	31	29.8
^a^Formal training obtained in healthcare management		
Certificate *(Formal)*	19	18.3
Diploma *(Formal)*	24	23.1
Degree *(Formal)*	32	30.8
None *(No formal)*	29	27.9
Informal training obtained in healthcare management		
In-service training	88	84.6
Mentoring	15	14.4
Non certified courses	1	1.0
Intention to attend healthcare mgt. Prog. within the next 5 years		
Yes	65	62.5
No	39	37.5

^a^implies variables in which some groups were merged to enhance further analysis

Table 3 displays the influence of managers’ socio demographics, hospital and health management characteristics had on attainment of formal training in health management. For socio demographic characteristics, age (*p* = .032) and academic qualification (*p* < .001) had significant influence on training while sex (*p* = .238) had no influence. In age, training was associated more to older managers: 45–60 years (77.1%) and 35–45 years (76.4%) than younger managers: 25–35 years (42.9%). In academic qualification, training was more among managers with higher degree (92.2%) than those with first degree (38.9%). Formal training refers to having the requisite training and qualification in hospital management and administration which many of the managers never had at the point of employment as this programme was not available in Nigerian Universities until recently. Given their background, many of them were forced to obtain both formal and informal trainings in hospital management and administration after having been employed as managers in their respective hospitals to enhance their performance. The programme in hospital management and administration is now being offered in Nigerian universities in the last ten years or so.

**Table 3:** Assessing the influence of managers’ socio demographic, hospital and health management characteristics on attainment of formal training in health management

	Training in Health Mgt.		Chi-Square	df	*p*-value
	Formal	No formal			
Age					
25–35 years	6(42.9)	8(57.1)	6.893	2	.032
35–45 years	42(76.4)	13(23.6)			
45–60 years	27(77.1)	8(22.9)			
Sex					
Male	45(68.2)	21(31.8)	1.390	1	.238
Female	30(78.9)	8(21.1)			
Academic qualifications					
1st Degree	14(38.9)	22(61.1)	33.207	1	< .001
Higher Degree	59(92.2)	5(7.8)			
Hospital type					
Private	19(43.2)	25(56.8)	32.365	3	< .001
Government	37(90.2)	4(9.8)			
Non-governmental	5(100.0)	0(0.0)			
Faith based	14(100.0)	0(0.0)			
No. of hospital bed					
25–50 beds	8(30.8)	18(69.2)	29.981	2	< .001
50–100 beds	46(88.5)	6(11.5)			
> 100 beds	21(80.8)	5(19.2)			
Number of staff					
Below 25	10(50.0)	10(50.0)	18.830	3	< .001
25–50	20(57.1)	15(42.9)			
50–100	32(88.9)	4(11.1)			
100 and above	13(100.0)	0(0.0)			
Current designation					
Administrative officer	23(92.0)	2(8.0)	16.944	3	.001
Hospital administrator	14(73.7)	5(26.3)			
CEO/Hospital director	7(36.8)	12(63.2)			
Medical director	31(75.6)	10(24.4)			
Experience in hospital mgt.					
Less than 2 years	20(83.3)	4(16.7)	2.076	2	.354
3–10 years	33(67.3)	16(32.7)			
> 10 years	22(71.0)	9(29.0)			
Informal training					
In-service training	64(72.7)	24(27.3)	–	–	1.000*
Mentoring	11(73.3)	4(26.7)			
Intention to attend within 5 years					
Yes	44(67.7)	21(32.3)	1.686	1	.194
No	31(79.5)	8(20.5)			

*Fishers Exact test was reported due small expected frequency

For hospital characteristics, hospital type (*p* < .001), number of hospital bed (*p* < .001) and number of staff (*p* < .001) had significant influence on training in health management. In hospital type, training was associated more to managers in faith-based (100.0%), non-governmental (100.0%) and government hospital (90.2%) than those in private hospital (43.2%). In number of hospital bed, training was more among those who had higher number of bed: 50–100 beds (88.5%) and above 100 beds (80.8%) than those with fewer beds: 25–50 beds (30.8%). Likewise in number of staff, training was associated to managers with more number of staff: 100 staff and above (100.0%) and 50–100 staff (88.9%) than those with fewer staff: 25–50 staff (57.1%) and below 25 staff (50.0%).

For health management characteristics, managers’ current designation had significant influence on training (*p* = .001), while experience in health management (*p* = .354), **i**nformal training type (*p* = 1.000) and intention status on whether to attend health management programme within 5 years (*p* = .194) had no influence. In designation, training was least among CEO/hospital directors (36.8%) and most among administrative officers (92.0%); training among medical directors (75.6%) and hospital administrators (73.7%) were middling though reasonable high.

Tables 4, 5, the logistic regression model [logit (being formally trained) = 2.667 + 1.353*age + 0.474*gender + 3.237*academic qualification – 1.469*hospital type + 0.818*(25–50 beds) + 0.706*(50–100 beds) – 1.206*(below 25 staff) + 0.014*(25–50 staff) – 0.495*hospital administrator – 2.065*CEO/hospital director – 0.311*medical director – 2.146*(3–10 years) – 3.498*(above 10 years) – 0.795*intention to attend] explained 62.1% (Nagelkerke R^2^) of the variation in managers’ health management training status (that is, whether a manager is formally trained or not). It also correctly predicted the status of 88.0% of persons. The omnibus test of model coefficients using the Chi-Square revealed that the model coefficients were significant, χ^2^ (14) = 55.803, *p* < .001.

**Table 4:** Logistic regression classification table, model summary and omnibus test of model coefficients on attainment of formal training in health management

Classification table					Model summary			Omnibus test of model coefficients		
Observed		Predicted								
		Training in health mgt.		% Correct	-2 Log likelihood	Cox & Snell R^2^	Nagelkerke R^2^	χ^2^	df	*p*-value
		No formal	Formal							
Training in health mgt.	No formal	19	8	70.4	60.849	.428	.621	55.803	14	< .001
	Formal	4	69	94.5						
Overall %				88.0						

The cut value is .500; *n* = 100

**Table 5:** Logistic regression model coefficients on attainment of formal training in health management

	B	S.E.	Wald	df	*p*-value	Exp(B)	95% C.I. for EXP(B)	
							Lower	Upper
Constant	2.667	2.112	1.594	1	.207	14.394		
^a^Age (< 45 years)	1.353	1.404	.929	1	.335	3.870	.247	60.639
Gender (Female)	.474	.867	.299	1	.585	1.606	.293	8.795
^a^Academic qual. (Higher degree)	3.237	1.200	7.282	1	.007*	25.463	2.426	267.311
^a^Hospital type (Non government)	−1.469	1.163	1.595	1	.207	.230	.024	2.249
Hospital bed			.405	2	.817			
25–50 beds	.818	1.580	.268	1	.605	2.265	.102	50.136
50–100 beds	.706	1.133	.388	1	.533	2.025	.220	18.655
^a^Hospital staff			1.396	2	.498			
Below 25 staff	−1.206	1.190	1.027	1	.311	.299	.029	3.084
25–50 staff	.014	1.042	.000	1	.989	1.014	.131	7.819
Current Designation			2.596	3	.458			
Hospital administrator	−.495	1.313	.142	1	.706	.609	.046	7.992
CEO/hospital director	−2.065	1.400	2.175	1	.140	.127	.008	1.972
Medical director	−.311	1.161	.072	1	.789	.733	.075	7.130
Experience in hosp. Mgt.			5.183	2	.075			
3–10 years	−2.146	1.001	4.596	1	.032	.117	.016	.832
Above 10 years	−3.498	1.919	3.325	1	.068	.030	.001	1.299
Intention to attend prog. (Yes)	−.795	1.047	.576	1	.448	.452	.058	3.517

Predictors: Age, Gender, Academic qualification, Hospital type, No. of hospital bed, No. of staff, Current designation, Experience in hospital management & Intention to attend healthcare mgt programme in next 5 years

Reference category: Age (< 45 years), gender (male), academic qual. (1st degree), hospital type (government), hospital bed (> 100 beds), hospital staff (> 50 staff), designation (admin officer), experience in hosp. Mgt (≤ 2 years), intention to attend healthcare mgt prog (no)

*implies significant predictors; ^a^implies variables in which some groups were merged to enhance logistic regression.

However, the Wald statistic indicated that the model coefficient of only academic qualification (*p* = .007) was significant. This implies that in predicting a manager who is formally trained in health management, holding other predictors constant, managers with higher degree had odds 25.5 times higher the odds of those with first degree.

For the coefficients of age (*p* = .335), gender (*p* = .585), hospital type (*p* = .207), number of hospital beds (*p* = .817), number of hospital staff (*p* = .498), current designation (*p* = .458), experience in hospital management (*p* = .075) and intention status to attend healthcare management programme within the next 5 years (*p* = .448), the Wald statistic revealed no significance. This implies that holding other predictors constant, the managers grouped by their age or gender had the same odds in being formally trained in health management; likewise when grouped by hospital type, number of hospital beds in their hospital, number of hospital staff in their hospital, current designation, experience in hospital management and intention status to attend healthcare management programme within the next 5 years.

## Discussion

The results show that majority of the respondent managers were aged between 35 and 45 years (52.9%). There were more males (63.5%) than females (36.5%) amongst them. A little above thirty four (34.6%) percent had bachelor’s degree, 27.9% had post graduate degree while 33.7% had master’s degree. Most of them were either in the private hospital (42.3%) or government hospital (39.4%). In number of staff, those who had 50–100 staff (34.6%) were more followed by those who had 25–50 staff (33.7%) while in the number of beds, it was those who had 50–100 beds in their hospital (50.0%) were in majority. Twenty four (24.0%) percent of them were administrative officers, 18.3% were hospital administrators and as well CEO/hospital directors while 39.4% were medical directors. Majority of the respondent managers as well have had 3–10 years of hospital management experience (47.1%). In formal healthcare management training, only few had no training (27.9%) meaning majority have had formal training while on the job, and in informal healthcare management training, all had obtained a form of training of which in-service training predominates (84.6%). Most of the administrators/managers also had the intention of attending healthcare management programme within the next five years (62.5%). Socio demographically, age (*p* = .032) and academic qualification (*p* < .001) had significant influence on training while sex (*p* = .238) had no influence. In age, training was associated more to older managers: 45–60 years (77.1%) and 35–45 years (76.4%) than younger managers: 25–35 years (42.9%). In academic qualification, training was more among managers with higher degree (92.2%) than those with first degree (38.9%). Also influencing managers’ training in healthcare management were hospital type (*p* < .001), number of hospital beds (*p* < .001) and number of staff (*p* < .001) which had significant influence on training in health management. In hospital type, training was associated more to managers in faith-based (100.0%), non-governmental (100.0%) and government hospitals (90.2%) than those in private hospitals (43.2%). In number of hospital beds, training was more among those who had higher number of bed: 50–100 beds (88.5%) and above 100 beds (80.8%) than those with fewer beds− 25-50 beds (30.8%). Likewise in number of staff, training was associated to hospitals with more number of staff: 100 staff and above (100.0%) and 50–100 staff (88.9%) than those with fewer staff: 25–50 staff (57.1%) and below 25 staff (50.0%). Management characteristic that also had influence on managers training in health management was managers’ current designation which had significant influence on health management training while years of experience in health management (*p* = .354), informal training type (*p* = 1.000) and intention status on whether to attend health management programme within 5 years (*p* = .194) had no influence. In designation, training was least among CEO/hospital directors (36.8%) and most among administrative officers (92.0%); training among medical directors (75.6%) and hospital administrators (73.7%) were middling though reasonable high. The logistic regression model [logit] explained 74.2% (Nagelkerke R^2^) of the variation in managers’ health management training status (that is, whether a manager is formally trained or not). It also correctly predicted the status of 89.0% of persons. The omnibus test of model coefficients using the Chi-Square revealed that the model coefficients were significant, χ^2^ (15) = 71.510, *p* < .001.

The high level of informal training by the respondents as in the forms of seminars and workshops could have been brought on by the desired need for cash benefits accruable to such trainings rather than the managerial technical benefits. It could have as well exposed the lack of capacity in healthcare management by the respondent managers. Management approval for informal training could be seen as an admittance of failure and the need to improve on the capacity of its workforce. Whatever it is, the bills for such trainings are usually paid up front or settled as soon as the beneficiaries returned back to the office by the management. Informal trainings are usually slated or reserved for friends and supporters of the administration. The materials and technical contents of the trainings are usually set aside by the beneficiaries as the administration may not ask for the application nor the evaluation of what has been leant for general institutional benefits. This explains the high level of informal training which invariably adds little value to the managerial technical competence of the beneficiaries. This situation on training is supported by [1] where it was noted that many management staff in Nigerian hospitals engage in patchy informal training not for the technical managerial benefits but for the accruable financial benefits thereafter. Mentoring training is hardly practiced as many in management positions do not see the need for it nor have substantial managerial experience to pass on to aspiring managers. The equally high level of formal training in health care management (certificate, diploma and degree) by the management staff exposes their need to grasp with the enormous responsibilities of managing hospitals which are very technical in nature. Majority of the managers never had the requisite managerial competence required of their position on employment and could only make it up by acquiring health management certificates, diplomas and degrees on employment. Formal managerial training actually meant outright enrollment into institutions of higher learning by the managers in human resource management, Institutional financial management and economics. These trainings were lacking in the curricula during their university attendance and could only be made up through additional formal trainings on employment to cope with the enormous responsibilities of hospital management. This situation is supported by [1] which shows that many health managers lack adequate training in providing strategies that enable prudent management of all the available resources and amenities for the proper functioning of the hospitals. This lack of formal training affects the performance of the health work force and accounts for the low productivity of practicing health managers as emphasized by [2–6] and could only be corrected through acquiring additional managerial formal trainings at the post graduate level. Institutional performance failures usually stem from lack of capacity of practicing managers who usually lack the necessary competence and skills needed to harness the available human and materials resources in their institutions especially in the Nigerian context. Lack of training affects the performance of the workforce and it is only when this is taken seriously would hospitals in this case realize their full performance potentials.

The high level of intention to attend such training in the future speaks about the realized need for such training and reflects admitted lack of knowledge and capacity by managers in managerial strategies. Even at that, some managers have not yet identified the need for such training as they do not see such training as their domain, and as such reasoned that it should be left to the economists and administrators [24]. This finding is supported by [15] that opined that Physicians may reason that their energy, not to mention their years of education and training, should be focused on patient care, research, and staying abreast of clinical advancements, whereas staff education should fall under the category of personnel and be the responsibility of practice managers. However, the need for training in hospital management as found in our work has been supported and emphasized by [7–11] who opined that continuing education and training have become part of the ongoing processes of organizational learning and permanent change, employee evaluation and career development. Organisational learning helps managers to improve their management skills and to learn new skills as opined by [7–11] which support our findings in that there was an express need by managers in our study to acquire additional trainings and capacity that stemmed from their lack of performance. The global trend has been an increasing demand for both formal and informal trainings for health organizations especially for physicians in leadership position as advanced by [7, 12–14] and this represents our position for hospitals of the future in Nigeria.

A relationship was rather found between respondents’ age and the attainment of formal training as the younger the age of the respondents, the lower the attainment of formal training. This goes to show that the younger managers are yet to uncover the need for formal managerial training since they are still new on the job compared to the older managers. It also goes to show that no immediate changes have been done to the curricula of healthcare providers especially in medical schools to reflect needed changes that inform formal managerial training. At the same time, it could be just that people who have been in the system for long would have served long enough to earn the opportunity to have further training as in the older managers as opposed to younger ones. Also small hospitals may not have enough resources to pay for management courses (even if they had the will), unlike the big ones (often state or federal) institutions with unspent budgets who can afford such training. This could also go to explain why younger managers who may be employed in smaller hospitals may be indisposed to acquiring such formal training.

It was also found that the higher the number of hospital beds, the greater the tendency to have acquired formal training according to the result. The number of hospital beds in Nigeria, most often reflects how large the hospital is. Most public hospitals have higher number of beds compared to private hospitals and are more likely to undertake the acquisition of formal trainings as managers are granted the opportunity to further their education either on part-time or full-time basis. Usually smaller and private hospitals hardly undertake the training of their staff nor do they grant study leaves for the completion of such trainings. For better performance of the health system, it is highly recommend that smaller and private health care organisations find a way of boosting the trainings of their managers in management, economics and human resource programmes.

Finally the result equally showed that, the higher the educational level of the health managers, the higher the attainment of formal training. This goes to show that those managers who must have acquired some level of higher education may have done so not in health managerial training reflecting the need for such training in aiding managers perform more effectively. This is supported by [2, 4, 24] which opined that health professionals function both as clinicians and managers in Nigeria, yet training in health services management missing in the curricula is critical to bridging the knowledge gap for health professionals to perform management functions effectively.

Our work has demonstrated the need for both immediate and long term solutions to the problem of lack of managerial training and capacity amongst health managers in Nigeria. And as such we do recommend short term courses in hospital management and economics in the short run and the inclusion of same in the curricula of healthcare providers while undertaking university programmes in specific health areas. Opportunities should also be made available to managers to enroll in hospital management programmes to forestall the lack of capacity being experienced by our hospital managers. The younger managers should be targeted the most since they are the least likely to engage in formal managerial training.

## Conclusion

Our work has established the critical need for both informal and formal trainings in health management for health care managers and for such to be applied to enable them effectively manage the enormous responsibilities of the health sector and hospitals in particular. Emphasis in training should be directed at younger managers who were least likely to acquire such trainings, the smaller and private hospitals that are less likely to encourage such trainings amongst their staff and the least educated among health managers who are less likely to acquire higher education in health management. Finally, training in management should be made a requirement for promotion along the management cadre in our health system and hospitals in particular to enable its application by managers with administrative responsibilities. More so, license renewal for physicians in management positions in our hospitals should require some training in hospital management to bridge the gap in their health management capacity.

## Additional files

Additional file 1: Data collection on predictors for managerial trainings. (XLSX 29 kb)

Additional file 2: Data on assessment of managerial trainings. (DOCX 16 kb)

## Abbreviations

CMD: Chief Medical Director
FCT: Federal Capital Territory
IVF: Invitro Fertilization
