# Supplementary material for: Assessing the predictors for training in management amongst hospital managers and chief executive officers: a cross-sectional study of hospitals in Abuja, Nigeria
Source: BMC Med Educ. 2018 Jun 14;18:138. doi: 10.1186/s12909-018-1230-2 (PMC6003084; doi:10.1186/s12909-018-1230-2)
Supplement: Supplementary file 2 — Data on assessment of managerial trainings. (DOCX 16 kb) [file 12909_2018_1230_MOESM2_ESM.docx]

**QUESTIONAIRE ONASSESSMENT OF MANAGERIAL COMPETENCIES IN HOSPITALS**

AN ASSESSMENT OF LEVELS MANAGERIAL COMPETENCIES OF HOSPITAL ADMINISTRATORS: A CASE STUDY OF HOSPITALS IN THE FEDERAL CAPITAL TERRITORY (FCT), ABUJA, NIGERIA.

This is purely an academic research in partial fulfillment for the award of postgraduate diploma in health economics, management and policy (HEMP) of the University of Nigeria, Nsukka.

Please answer the following questions by making a cross in the appropriate block.

1. Biographic details
   1. Gender: Male ( ) Female ( )
   2. Age: 25-35 ( ) 35-45 ( ) 45-60 ( )
   3. Type of hospital: private, government, Non-governmental, faith based

( ) ( ) ( ) ( )

- 1. Hospital beds 25-50 ( ) 50-100 ( ) above 100 ( )
  2. Current designation

(a) Administrative officer ( )

(b) Hospital administrator ( )

(c) CEO/hospital director ( )

(d) Medical director ( )

(e) Others (please specify) -----------------

- 1. Experience in hospital management

1. Less than two years ( )
2. 3-10 years ( )
3. Above 10 years ( )
   1. Academic qualifications

(a) Bachelor’s degree ( )

(b) Post graduate diploma ( )

(c) Master’s degree & higher ( )

(d) Others (please specify) ( ) ----------------------------------------

- 1. Have you had any formal training in healthcare management?

1. Certificate ( )

(b) Diploma ( )

(c) Degree ( )

(d) None ( )

(e) Others (please specify) ( ) -----------------------------------------

1.9 Have you had any informal training in healthcare management?

(a) In-service training (workshops, seminars etc) ( )

(b) Mentoring ( )

(c) Non certified courses ( )

(d) Others (please specify) ( ) ----------------------------

1.10 Do you intend to attend any health care management programme within the next five years?

(a) Yes ( )

(b) No ( )

If yes please specify ---------------------------------------------------------------------------

2.0 Number of staff in the hospital

(a) Below 24 ( )

(b) 25-50 ( )

(c) 50-100 ( )

(d) 100 and above ( )

3.0 DEVELOPED ABILITIES:

Please indicate your level of competence with the following skills:

Very poor poor good excellent

3.1 program & project mgt ( ) ( ) ( ) ( )

3.2 financial mgt ( ) ( ) ( ) ( )

3.3 service delivery & innovation ( ) ( ) ( ) ( )

3.4 change mgt ( ) ( ) ( ) ( )

3.5 knowledge mgt ( ) ( ) ( ) ( )

3.6 HR empowerment ( ) ( ) ( ) ( )

3.7 communication skills ( ) ( ) ( ) ( )

3.8 honesty & integrity ( ) ( ) ( ) ( )

3.9 leadership& Influence ( ) ( ) ( ) ( )

3.10 Clients care mgt ( ) ( ) ( ) ( )

COMPETENCIES IN MANAGERIAL SKILLS:

4.0 PLANNING SKILLS:

(a) Programme planning ( ) ( ) ( ) ( )

(b) Preparation of a strategic plan ( ) ( ) ( ) ( )

(c) Creating a vision for your hospital ( ) ( ) ( ) ( )

4.1 LEADING SKILLS:

(a) Team management ( ) ( ) ( ) ( )

(b) Communicating organizational goals ( ) ( ) ( ) ( )

(c) Managing conflicts ( ) ( ) ( ) ( )

(d) Motivating employees ( ) ( ) ( ) ( )

(e) Labour relations ( ) ( ) ( ) ( )

(f) Setting organizational culture ( ) ( ) ( ) ( )

4.2 CONTROLLING SKILLS:

(a) Measurement of organizational performance ( ) ( ) ( ) ( )

(b) Assessing the quality of hospital care ( ) ( ) ( ) ( )

(c) Evaluating health services delivery progress ( ) ( ) ( ) ( )

(d) Financial performance evaluation ( ) ( ) ( ) ( )

(e) Assessing patients satisfaction ( ) ( ) ( ) ( )

(f) Implementing health quality improvement system ( ) ( ) ( ) ( )

(g) Nursing quality management ( ) ( ) ( ) ( )

(h) Providing feedback to patients & staff ( ) ( ) ( ) ( )

4.3 ORGANISATION SKILLS:

(a) Structure health service organizations ( ) ( ) ( ) ( )

(b) Budgeting ( ) ( ) ( ) ( )

(c) Resource planning ( ) ( ) ( ) ( )

(d) Human resource planning ( ) ( ) ( ) ( )

(e) Use health technology ( ) ( ) ( ) ( )

(f) Performance appraisal ( ) ( ) ( ) ( )

(g) Organizing nursing training ( ) ( ) ( ) ( )

(h) Allocation of financial resources ( ) ( ) ( ) ( )

4.4 SELF ASSESSMENT

(a) Time management ( ) ( ) ( ) ( )

(b) Acting independently ( ) ( ) ( ) ( )

(c) Awareness of personal weakness and strength ( ) ( ) ( ) ( )

(d) Balancing work and life issues ( ) ( ) ( ) ( )

(e) Ability to learn from experience ( ) ( ) ( ) ( )

(f) Self-development ( ) ( ) ( ) ( )
